# Commercially available SARS-CoV-2 RT-qPCR diagnostic tests need obligatory internal validation

**DOI:** 10.1038/s41598-023-34220-w

**Published:** 2023-04-28

**Authors:** Katarzyna Linkowska, Tomasz Bogiel, Katarzyna Lamperska, Andrzej Marszałek, Jarosław Starzyński, Łukasz Szylberg, Aleksandra Szwed-Kowalska, Małgorzata Pawłowska, Tomasz Grzybowski

**Affiliations:** 1 Department of Forensic Medicine, Collegium Medicum of the Nicolaus Copernicus University, Curie-Skłodowskiej Str. 9, 85-094 Bydgoszcz, Poland; 2 Department of Microbiology, Collegium Medicum of the Nicolaus Copernicus University, Bydgoszcz, Poland; 3 Laboratory of Cancer Genetics, Greater Poland Cancer Centre, Poznań, Poland; 4 Department of Oncologic Pathology and Prophylaxis, Greater Poland Cancer Centre, Poznań University of Medical Sciences, Poznań, Poland; 5 Department of Tumor Pathology and Pathomorphology, Oncology Centre - Prof. Franciszek Łukaszczyk Memorial Hospital, Bydgoszcz, Poland; 6 Department of Perinatology, Gynaecology and Gynaecologic Oncology, Collegium Medicum of the Nicolaus Copernicus University, Bydgoszcz, Poland; 7 Department of Laboratory Diagnostics, The Tadeusz Browicz Provincial Hospital for Infectious Diseases and Observation, Bydgoszcz, Poland; 8 Department of Infectious Diseases and Hepathology, Collegium Medicum of the Nicolaus Copernicus University, Bydgoszcz, Poland

**Keywords:** PCR-based techniques, Infectious-disease diagnostics

## Abstract

Although infection with severe acute respiratory syndrome coronavirus 2 (SARS CoV-2) does not appear to be as serious a threat to public health as it was in 2020–2021, the increased transmissibility of multiple Omicron descendants may constitute a continuous challenge for health care systems, and reliable detection of new variants is still imperative. This study evaluates the performance of three SARS-CoV-2 diagnostic tests: Novel Coronavirus (2019-nCoV) Real Time Multiplex RT-PCR Kit (Liferiver); Vitassay qPCR SARS-CoV-2 (Vitaassay) and TaqPath COVID‑19 CE-IVD RT-PCR Kit (Thermo Fisher Scientific). The analytical sensitivity of the assays as well as their specificity were determined with the use of synthetic nucleic acid standards and clinical samples. All assays appeared to be 100% specific for SARS-CoV-2 RNA in general and the Omicron variant in particular. The LOD determined during this validation was 10 viral RNA copies/reaction for Liferiver and TaqPath and 100 viral RNA copies for Vitassay. We cannot exclude that the LOD for the Vitassay might be lower and close to the manufacturer's declared value of ≥ 20 genome copies/reaction, as we obtained 90% positive results for 10 viral RNA copies/reaction. Mean Ct values at the concentration of 10 viral RNA copies/reaction for the Liferiver, Vitassay and TaqPath kits (35, 37 and 33, respectively) were significantly lower than the cutoff values declared by the manufacturers (≤ 41, ≤ 40 and ≤ 37, respectively). We suggest reporting outcomes based on LOD and cutoff Ct values determined during internal validation rather than those declared by the assays’ producers.

## Introduction

The coronavirus disease 2019 (COVID-19) pandemic has forced the scientific community to rapidly develop highly reliable, high-throughput methods dedicated to fast and accurate diagnosis of the condition. Indeed, validated and accurate laboratory testing for severe acute respiratory syndrome coronavirus 2 (SARS-CoV-2) is a crucial part of the timely management of COVID-19, supporting the clinical decision-making process for infection control and consequently containing the pandemic.

The gold standard in medical diagnosis of SARS-CoV-2 virus is molecular assays based on amplification of viral RNA^1^, with tests based on reverse transcription and quantitative real-time PCR (RT-qPCR) being most widely implemented. As of October 2022, a total of 592 commercially available RT-qPCR tests targeting viral RNA were listed on the FIND website^2^. Although it is wise to assume that most of these tests were developmentally validated by their producers, the data on independent, internal validation of particular assays by their users or other interested parties are still very limited^3–9^. Moreover, existing assays, which are based on different primer–probe sets, are characterized by different analytical specificities and sensitivities, and this may affect their diagnostic value and cause different outcomes to be partially incomparable^10,11^. In addition, some pitfalls resulting from low-level contamination of commercially available assays have been reported, which can be detected only during thorough internal validation^12^. Most importantly, RT-qPCR SARS-CoV-2 assays, as with any other diagnostic tests, should be subjected to internal validation in medical diagnostic laboratories, in line with ISO 15,189 requirements or general ISO 17,025 standards. Although that “common sense” principle had been unequivocally articulated at the beginning of extensive SARS-CoV-2 testing^13^, its actual realization in many national testing policies was far from perfect^14^. Consequently, millions of tests were performed with the use of unvalidated or incompletely validated diagnostic assays, potentially undermining public understanding and support for testing and discrediting science in general. Conversely, as test outcomes might have significant administrative consequences and influence the epidemic’s containment policy, the methodology of testing should not involve doubts or controversies.

After several waves of infections during the COVID-19 pandemic, our understanding of SARS-CoV-2 biology, its variability and its ability to infect and spread has increased. Additionally, our knowledge of the social, economic and health care implications of the COVID-19 diagnostic policy has improved. Under these circumstances, it seems important to re-evaluate the currently available diagnostic tests, especially in terms of their analytical sensitivity and specificity toward new variants of the virus. Moreover, considering the internal validation results, improving interpretation of diagnostic outcomes is warranted. Taken together, such measures would better address the current epidemiological situation.

Based on these assumptions, the aim of the study was to validate and compare three RT-qPCR tests intended for qualitative detection of nucleic acids from SARS-CoV-2 in upper respiratory and bronchoalveolar lavage (BAL) specimens from individuals suspected of having COVID-19. We chose three SARS-CoV-2 diagnostic tests: Novel Coronavirus (2019-nCoV) Real Time Multiplex RT-PCR Kit (Liferiver Shanghai ZJ Bio-Tech Co., Ltd., Shanghai, China); Vitassay qPCR SARS-CoV-2 (Vitassay Health care, S.L.U., Huesca, Spain) and TaqPath COVID‑19 CE-IVD RT-PCR Kit (Thermo Fisher Scientific, Waltham, MA, USA), which are widely used in various parts of the world. Unfortunately, we did not have an access to the CDC’s RT- qPCR test for the SARS-CoV-2 to perform it as one of the methods for the validation.

## Materials and methods

### RT-qPCR kits for COVID-19 diagnosis used in the present study

#### TaqPath COVID‑19 CE-IVD RT-PCR Kit (hereafter referred to as TaqPath)

TaqPath was performed by real-time PCR, according to the manufacturer’s protocol for ≤ 200 µl sample input volume using ViiA 7 Real-Time PCR System (Applied Biosystems, Foster City, California, USA). The assay targets three genomic regions of SARS-CoV-2: the ORF 1ab gene, N gene and S gene. The PCR contained 5 µl of purified sample RNA, 6.25 µl of TaqPath 1‑Step Multiplex Master Mix, and 1.25 µl of COVID-19 Real Time PCR Assay Multiplex in a final volume of 25 µl. The amplification conditions were 2 min at 25 °C; 10 min at 53 °C; and 40 cycles of 95 °C for 3 s and 60 °C for 30 s. The cycle number at which the fluorescent signal of the reaction crosses the threshold is referred to as the threshold cycle (Ct). The Ct value is inversely related to the starting amount of target DNA. The Ct cutoff values for assay targets are used for interpretation of the results. The manufacturer’s Ct cutoff values for viral targets were ≤ 37. The TaqPath kit includes an RNA phage control (MS2) to verify the efficiency of the sample preparation and the absence of inhibitors in the PCR. As the clinical samples used in this study were residues from diagnostic analysis, an MS2 control was not used. However, positive and negative controls were included in each assay.

#### Vitassay qPCR SARS-CoV-2 Kit (hereafter referred to as Vitassay)

Vitassay was performed by real-time PCR according to the manufacturer’s protocol for the ready-to-use test, which contains all the necessary reagents in a stabilized format in each well of the plate. Reactions were performed using ViiA 7 Real-Time PCR System. The assay targets two specific fragments of the SARS-CoV-2 genome: the ORF 1ab gene and the N gene. To each well with real-time PCR reagents, 15 µl resuspension buffer and 5 µl purified sample RNA were added. The amplification conditions were 15 min at 45 °C and 45 cycles of 95 °C for 10 s and 60 °C for 50 s. The producer’s Ct cutoff values for viral targets during interpretation of the results were ≤ 40. To confirm the appropriate performance of the technique, an internal control (IC), as well as positive and negative controls, were included in each assay.

#### Novel Coronavirus (2019-nCoV) Real Time Multiplex RT-PCR Kit (hereafter referred to as Liferiver)

Liferiver was performed by real-time PCR, according to the manufacturer’s protocol using ViiA 7 Real-Time PCR System. The assay targets three genomic regions of SARS-CoV-2: the ORF 1ab gene, N gene and E gene. The PCR comprised 5 µl of purified sample RNA, 19 µl Super Mix and 1 µl RT-PCR Enzyme Mix in a final volume of 25 µl. The amplification conditions were 10 min at 45 °C; 3 min at 95 °C; and 45 cycles of 95 °C for 15 s and 58 °C for 30 s. The producer’s Ct cutoff values for viral targets during interpretation of the results were ≤ 41. The Liferiver kit includes an internal control (IC), which is a plasmid containing a nontargeted RNA fragment, to evaluate extraction efficiency and the absence of PCR inhibitors. Moreover, both positive and negative controls were included in each assay.

### Analytical sensitivity

Quantitative Synthetic SARS-CoV-2 RNA (cat. number ATCC-VR-3276SD, LGC) was used as a reference material to determine the efficiency of the PCR and the sensitivity of individual tests by testing tenfold dilutions (10^6^–10^1^ viral RNA copies per reaction) in four replicates. Analytical sensitivity is defined herein as the limit of RNA detection (LOD). LOD is the lowest tested viral copy number for which 95% of the replicates are detected (Ct value is ≤ Ct cutoff value for the assay targets). We tested 10^2^–10^0^ viral RNA copies per reaction for twenty replicates to determine the LOD of each kit. The N gene, which is common to all kits, was used for comparisons.

### Analytical specificity

The specificity of the kits as well as the potential cross-reactions with other pathogens residing in mucous membranes of the respiratory tract or causing respiratory infections, including *Neisseria spp., Acinetobacter baumannii, Candida glabrata, Staphylococcus epidermidis, Haemophilus parainfluenzae*, *Escherichia coli, beta-hemolytic streptococci, Pseudomonas aeruginosa,* RSV and influenza virus, were tested by analyzing anonymised nucleic acid extracts from fifteen clinical samples in which the presence of at least one of the mentioned pathogens was confirmed. In addition, the analytical specificity of the kits was assessed using the molecular standards of the following pathogens: human rhinovirus 16 and 17; human coronavirus NL63, HKU1, 229E; enterovirus D68; influenza A and B virus; human adenovirus 1, 2, 4 and 31; and human parainfluenzavirus 2 and 3 (Table 1). The impact of other interfering substances was tested by analyzing the anonymised nucleic acid extracts from buccal swabs of eight healthy individuals.

**Table 1:** List of commercially available molecular standards (nucleic acid solutions) used in analysis of the specificity of the three studied RT-qPCR assays.

Reference material	Cat. Number
Quantitative Genomic RNA from Human rhinovirus 16 strain 11,757	ATCC-VR-283DQ
Genomic RNA from Human rhinovirus 17 strain 33,342	ATCC-VR-1663D
Quantitative Synthetic Human coronavirus NL63 RNA	ATCC-VR-3263SD
Quantitative Synthetic Human coronavirus HKU1 RNA	ATCC-VR-3262SD
Quantitative Genomic RNA from Human coronavirus 229E	ATCC-VR-740DQ
Genomic RNA from Enterovirus D68 strain US/KY/14–18,953	ATCC-VR-1825D
Genomic RNA from Influenza A virus (H1N1) strain A/Virginia/ATCC1/2009	ATCC-VR-1736D
Quantitative Genomic RNA from Influenza A virus H1N1 strain A/California/07/2009 (H1N1)pdm09	ATCC-VR-1894DQ
Quantitative Genomic RNA from Influenza B virus (Yamagata Lineage) strain B/Wisconsin/1/2010 BX-41A	ATCC-VR-1885DQ
Quantitative Genomic RNA from Influenza B virus	ATCC-VR-1804DQ
Genomic DNA from Human adenovirus 1 strain Adenoid 71	ATCC-VR-1D
Quantitative Genomic DNA from Human adenovirus 2 strain Adenoid 6	ATCC-VR-846DQ
Quantitative Genomic DNA from Human adenovirus 4 strain RI-67	ATCC-VR-1572DQ
Genomic DNA from Human adenovirus 31 strain 1315	ATCC-VR-1109D
Quantitative Genomic RNA from Human parainfluenza virus 2 strain Greer	ATCC-VR-92DQ
Quantitative Genomic RNA from Human parainfluenzavirus 3 strain C 243	ATCC-VR-93DQ
Quantitative genomic RNA from Severe acute respiratory syndrome-related coronavirus 2 strain 2019-nCoV/USA-WA1/2020	ATCC-VR-1986D

### Confirmation of the results on clinical samples

A total of 132 residual, randomly selected RNA samples previously subjected to routine SARS-CoV-2 diagnostics were obtained from the Department of Microbiology (Nicolaus Copernicus University, Bydgoszcz, Poland), Department of Laboratory Diagnostics (The Tadeusz Browicz Provincial Hospital for Infectious Diseases and Observation, Bydgoszcz, Poland), Department of Tumor Pathology and Pathomorphology (Oncology Centre—Prof. Franciszek Łukaszczyk Memorial Hospital, Bydgoszcz, Poland) and Department of Oncologic Pathology and Prophylaxis (Greater Poland Cancer Centre, Poznań, Poland). The RNA samples had been extracted from nasopharyngeal swabs collected by trained and qualified personnel and preserved in virus transport and preservation medium (VTM). The samples had been completely anonymized before subjecting them to the study. The study was approved by the Bioethics Committee of Nicolaus Copernicus University (consent numbers KB 168/2021 and 169/2021). The Bioethics Committee of Nicolaus Copernicus University granted a waiver of informed consent. All methods were carried out in accordance with relevant guidelines and regulations.

### SARS-CoV-2 sequencing

Fourteen clinical samples with Ct ≤ 28 were randomly selected and subjected to NGS sequencing. The NGS approach by ISeq 100 System (Illumina, San Diego, CA, USA) provided 2 × 151 bp read data. Illumina COVIDSeq Test (Illumina) was used according to the manufacturer’s instructions. The analysis was performed with DRAGEN COVID Lineage Pipeline. Then, genomic lineages were classified and designated using Pangolin nomenclature^15^.

### Statistical analysis

The χ^2^ test was used to calculate significant differences between assay results. The significance level for statistical tests was 0.05. The statistical calculations, including the correlation coefficient (R^2^) determination, were performed using the Statistica package v.12.5 (Statsoft).

## Results

### Results of the validation study

**Figure 1:**
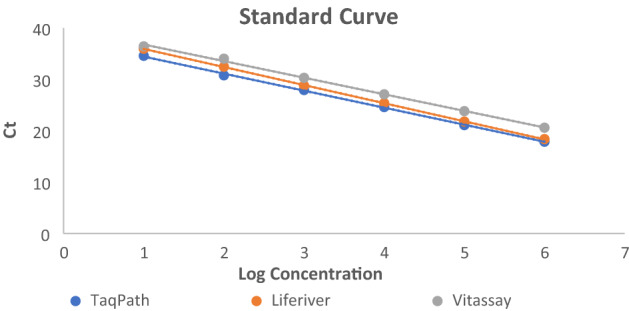
Standard curves established for Novel Coronavirus (2019-nCoV) Real Time Multiplex RT-PCR Kit (Liferiver), Vitassay qPCR SARS-CoV-2 (Vitassay) and TaqPath COVID‑19 CE-IVD RT-PCR Kit (Thermo Fisher Scientific).

Tenfold serial dilutions of viral RNA standard (10^6^–10^1^ viral RNA copies per reaction) were used to establish standard curves for assessing reaction efficiency (Fig. 1). We found that the efficiencies of particular tests were between 92 and 103% (Table 2), which matches the criteria of an efficient RT-qPCR assay. Similarly, the correlation coefficient (R^2^) value of each kit > 0.99 provides good confidence for the test results (Table 2). The Ct values with which the expected linear dilution series would cross the y-intercept when testing one viral RNA copy were equal to the cutoff Ct values for the Vitassay kit (Table 2). For the TaqPath and Liferiver kits, the y-intercept Ct values were one and two, respectively, Cts lower than the cutoff values declared for these tests (Table 2).

**Table 2:** RT-qPCR amplification parameters for Novel Coronavirus (2019-nCoV) Real Time Multiplex RT-PCR Kit (Liferiver), Vitassay qPCR SARS-CoV-2 (Vitassay) and TaqPath COVID‑19 CE-IVD RT-PCR Kit (Thermo Fisher Scientific).

	Efficiency (%)	R^2^	y-intercept	Mean Ct (SD) for 10^6^ – 10^1^ viral RNA copies per reaction					
				10^6^	10^5^	10^4^	10^3^	10^2^	10^1^
Liferiver	92	0.999	39	18.251 (0.045)	21.640 (0.135)	25.226 (0.171)	28.612 (0.242)	32.306 (0.143)	35.794 (0.173)
Vitassay	103	0.996	40	20.847 (0.220)	24.041 (0.069)	27.440 (0.207)	30.606 (0.174)	34.388 (0.120)	36.724 (0.421)
TaqPath	100	0.998	36	16.298 (0.162)	19.509 (0.423)	23.130 (0.119)	26.584 (0.163)	29.495 (0.144)	32.858 (0.201)

R^2^—the correlation coefficient, SD—standard deviation.

The manufacturers’ declared LOD values of the Liferiver, Vitassay and TaqPath kits were ≥ 25, ≥ 20 and ≥ 10 genome copies/reaction, respectively. Our data showed that Liferiver and TaqPath were capable of detecting the virus in all samples (100% positive results) at a concentration of 10 viral RNA copies/reaction. For Vitassay, we obtained 90% positive results at 10 viral RNA copies/reaction and 100% positive results at 100 virial copy/reaction (Table 3). However, it is worth noting that the mean Ct values for 10 viral RNA copies per reaction were 2.8–5.5 Cts lower than the cutoff Ct values declared by the manufacturers for individual tests. All kits were 100% sensitive for SARS-CoV-2 detection at 100 viral RNA copies/reaction and 25–50% sensitive at 1 viral RNA copy/reaction.

**Table 3:** Sensitivity of the studied RT-qPCR assays determined by analysis of twenty replicates of samples representing 1–100 copies of viral RNA per reaction.

	Number of positive results (N = 20)		
	1 copy/rxn	10 copies/rxn	100 copies/rxn
Liferiver	10 (mean Ct = 37.272)	20 (mean Ct = 35.476)	20 (mean Ct = 32.283)
Vitassay	5 (mean Ct = 38.758)	18 (mean Ct = 37.218)	20 (mean Ct = 34.307)
TaqPath	7 (mean Ct = 36.035)	20 (mean Ct = 32.971)	20 (mean Ct = 29.586)

Mean Ct values corresponding to the respective copy numbers are given in brackets.

Analysis of specificity and cross-reactivity with the use of the microorganisms listed in Table 1 revealed negative results for all three kits. Similarly, in clinical samples containing at least one pathogen causing respiratory infections, no cross-reactivity was observed between any of the following: *Neisseria spp., Acinetobacter baumannii, Candida glabrata, Staphylococcus epidermidis,* Haemophilus parainfluenzae, *Escherichia coli, Beta-hemolytic streptococci, Pseudomonas aeruginosa*, RSV, and influenza virus. When using buccal swabs from eight healthy persons, no RT-qPCR amplification was observed for any of the tested kits. These findings suggest that there is no cross-reactivity between the tested assays and host or other possible microbial nucleic acids present in swabs from non-COVID individuals.

### Verification of assay results using clinical samples

A total of 132 patient samples were randomly selected and tested with the use of Liferiver, Vitassay and TaqPath kits (Table 4). There were no significant differences between the number of positive and negative results obtained when using the different tests (*p* = 0.8278). Results positive in two of the three tests were considered true positives. Similarly, results that were negative in two of the three tests were considered true negatives. Based on these criteria, we observed one false-positive sample obtained using Liferiver. There were two false-negatives: one using TaqPath and one using Vitassay. Sensitivity, as defined as the proportion of people with the disease who tested positive compared to the number of all the people infected with the virus, regardless of test result, and specificity, measured as the proportion of healthy people who tested negative compared to the total number of people not infected with the virus, are shown in Table 4.

**Table 4:** Differences in analytical sensitivity and specificity between the studied SARS-CoV-2 kits in clinical samples.

	Results			Sensitivity (%)	Specificity (%)
	Positive	Negative	Inconclusive		
Liferiver	86	45	1	100	97.8
Vitassay	82	48	2	98.8	100
TaqPath	82	50	0	98.8	100

For Liferiver, the result for one sample was interpreted as inconclusive. For this sample, a signal for gene E was detected (Ct = 37); the ORF1ab and N genes were undetermined. Two samples were classified as inconclusive with Vitassay. For the first sample, the signal for the ORF1ab gene was positive (Ct = 38), but the signal for the N gene was negative; for the second sample, signals were positive for *N* (Ct = 36) and negative for ORF1ab. These inconclusive results may be due to amplification failure, which is very likely at high Ct values. For results with a positive signal for only one gene, mutation in target genes or infection with other coronaviruses cannot be excluded. Unfortunately, all samples with inconclusive results did not meet viral load requirements to be characterized by NGS sequencing.

### NGS sequencing results

Fourteen samples with Ct ≤ 28 were randomly selected and subjected to genomic characterization by NGS sequencing. Four of them showed a negative signal for the S gene in the TaqPath COVID-19 diagnostic test, the condition referred to as S gene target failure (SGTF). All samples were assessed as Omicron variants of concern (Table 5).

**Table 5:** Results for NGS sequencing of 14 randomly selected RNA specimens.

Sample	Lineage	clade	VOC
**1**	BA.3	21M	Omicron
2	BA.2	21L	Omicron
3	BA.2	21L	Omicron
**4**	BA.1	21K	Omicron
5	BA.2	21L	Omicron
6	BA.2	21L	Omicron
7	BA.2	21L	Omicron
**8**	BA.1	21K	Omicron
9	BA.2	21L	Omicron
10	BA.2	21L	Omicron
**11**	BA.1	21K	Omicron
12	BA.2	21L	Omicron
13	BA.2	21L	Omicron
14	BA.2	21L	Omicron

Samples with the 69–70 amino acid deletion in the spike protein, presenting the S Gene Target Failure (SGTF) detection pattern in the TaqPath COVID-19 assay, are marked in bold.

## Discussion

Although infection with SARS CoV-2 does not appear to be such a serious threat to public health as it was 1–1.5 years ago, the increased transmissibility of Omicron and its descendants may still constitute a challenge for health care systems in upcoming months of autumn and winter^16^. Omicron carries more than 50 mutations relative to the ancestral Wuhan virus, of which over 30 amino acid changes concern the spike protein. Some of these mutations lead to increased transmissibility, higher viral binding affinity, and higher immune evasion^17^. After its initial discovery in November 2021, multiple Omicron sublineages have emerged (Pango lineages BA.1, BA.2, BA.3, BA.4, BA.5, and descendants thereof)^18–21^. Some of the newly emerging sublineages that are on the rise in different populations are believed to neutralize antibodies from previous infection and vaccination to an extreme degree^16^. Considering this, reliable detection of new virus variants is still imperative.

The scientific community is currently almost exclusively focused on test sensitivity, a measure of how well an individual assay can detect viral RNA molecules. Today, there are many different diagnostic methods dedicated to detection of SARS-CoV-2. Rapid antigen tests and colorimetric sensing loop-mediated isothermal amplification (RT-LAMP) methods are fast, easy to use and do not require skilled personnel and specialized infrastructure. However, they are less sensitive than RT-qPCR^22–24^. Moreover, RT-qPCR is a technique that allows for detection of not only the presence of the virus but also specific mutations relevant to the disease severity, transmission capacity, evolution of the virus and vaccine efficiency^23,25–28^. Therefore, RT-qPCR is still the main diagnostic method. Nonetheless, how a test is being used under specific conditions of particular laboratories is important, and the level of performance and limitations of these assays should be taken into account^13^. In this respect, it is worth noting that the cutoff Ct values declared by the test manufacturers are based on different experimental conditions, and lack of clinical verification is not uncommon^14^. Therefore, before starting SARS-CoV-2 RNA diagnostics, a laboratory should first evaluate the performance of the kit to be used.

Because a lab operating in the official public health care system should prove that all its diagnostic methods are appropriate for the intended use, internal validation is also required for commercial RT-qPCR kits, even if these assays were previously developmentally validated and/or formally approved, depending on regulations in particular countries. This study provides the results of internal validation and comparison of three RT-qPCR assays for SARS-CoV-2 RNA testing. Our findings show high similarity among these kits in terms of their analytical sensitivities and specificities for SARS-CoV-2 detection, which indicates that the outcomes of the Liferiver, Vitassay and TaqPath assays are highly comparable.

Moreover, our study confirmed the high specificity of all tested kits for the Omicron variant, which is characterized by a large number of mutations relative to the ancestral virus. Mutations in the target genes of the assay did not affect the amplification, except for samples with the 69–70 deletion in the spike protein, which presented the S Gene Target Failure (SGTF) detection pattern by the TaqPath kit. NGS confirmed that this mutation is present in BA.1 (21 K) and BA.3 but not in the BA.2 (21L) lineage^19^.

The discordances found in our study were mainly regarding samples with low positivity signals (Ct > 35) and frequently in only one of the two or three genes included in the tests. However, we detected a low rate of inconclusive or false-positive/negative results. Such discrepancies prompt reflection on cutoff Ct values. Additionally, validation revealed differences in the Ct values for the standard samples when using different diagnostic kits, which confirmed that the Ct value varies with different amplification strategies and laboratory equipment^10^. A critical assessment of various published studies on RT-qPCR assays used for SARS-CoV-2 diagnostics with their different indicators of positivity, i.e., Ct cutoff values, was provided by Sule and Oluwayelu^29^. They reported that Ct values of 25 to 28 were usually appropriate but that values > 28 might indicate doubtful outcomes.

The theoretical limit of detection (LOD) of qPCR is at an average concentration of three target molecules per reaction volume^30^. However, because of the noise contributed by sampling, extraction and RT-qPCR efficiency, the LOD in practice can be substantially higher^30^. The LOD determined during our validation was 10 viral RNA copies/reaction for Liferiver and TaqPath and 100 viral RNA copies for Vitassay. We cannot exclude that the LOD for the Vitassay might be lower and close to the manufacturer's declared value of ≥ 20 genome copies/reaction, as we obtained 90% positive results for 10 viral RNA copies/reaction. Nevertheless, we did not perform analysis on 20 viral RNA copies/reaction during our validation study. We demonstrated that the Ct values at a concentration of 10 viral RNA copies/reaction in the Liferiver, Vitassay and TaqPath kits were significantly lower than the cutoff values declared by the manufacturers, which were ≤ 41, ≤ 40 and ≤ 37, respectively. Moreover, the Ct values for 1 viral RNA copy/reaction (37, 39 and 36, respectively) were also lower than the cutoff values for all kits. It should be emphasized that for 1 viral RNA copy/reaction, positive results ranged from only 25% for the Vitassay to 50% for Liferiver. This raises the question of what is actually detected at higher Ct values and suggests lowering the cutoff value. On the other hand, lower cutoff values may increase the false-negative results.

However, shifting the cutoff value specified by the manufacturer to the value for 1 viral RNA copy/reaction determined during the internal validation does not constitute a risk of virus spread because patients with such low results are not capable of transmitting infectious virus particles. In this respect, multiple studies have established thresholds for the presence of infectious SARS-CoV-2, as assessed by isolation of culture-competent SARS-CoV-2 in cell lines. Bullard et al. revealed that infectivity (as defined by growth in cell culture) was significantly reduced when RT-PCR Ct values are > 24. For every 1-unit increase in Ct, the odds ratio for infectivity decreased by 32%^31^. Additionally, Wölfel et al. reported that the success of virus isolation depends on viral load, with samples containing < 10^6^ copies per ml never yielding an isolate^32^. Similarly, van Kampen et al. found that the probability of isolating infectious virus was less than 5% when the viral RNA load was below 6.63 Log10 RNA copies/ml^33^, and Marot et al. showed that no isolate was recovered when the viral load was below 5.83 Log10 RNA copies/ml^34^. Conversely, other studies have revealed that culturing SARS-CoV-2 is possible with samples containing significantly less than the previously claimed culturing threshold of 10^6^ genome equivalents^35,36^. Our study showed that the mean Ct values for 6.3 Log10 RNA copies/ml (which corresponds to the quantity 10^4^ copies/reaction) for Liferiver, Vitassay and TaqPath were approximately 25, 27 and 23, respectively; these values are well below the cutoff values specified by the manufacturers. Therefore, the use of a cutoff point at the level established in the validation process carries relatively low risks in the context of patient infectivity and spread of the pathogen.

Nevertheless, positive results with high Ct (low viral loads) can be seen in the early stages of infection before the patient becomes capable of transmission or late in infection when the risk of transmission is low^36^. Additionally, we cannot exclude that a high Ct may be due to inadequately collected or degraded samples. Therefore, the Ct for a swab taken at a single point in time is not a good indicator of a person's infectivity. For this reason, persons with Ct values between the cutoff values determined in the laboratory and those specified by the manufacturer of the RT-qPCR kit should be retested to determine the stage of illness.

Many papers illustrating the influence of sample type or storage conditions on RT-qPCR results have been published, sometimes with opposing conclusions^37–40^. Therefore, it is reasonable for a laboratory to identify and take into account all factors that may affect results in the validation process before introducing the method into routine practice.

In summary, validation of the method to determine the LOD and cutoff Ct value is important in the context of infectivity and the need for isolating patients. Considering the results of this study, it seems unjustified to rely strictly on the values determined by assay producers, irrespective of the different conditions of particular diagnostic laboratories. Instead, it seems reasonable to define cutoff values based on internal validation. As the results of this validation show, such a pragmatic approach would generally lessen the social and economic impact of the pandemic.

## Data Availability

The data for this study have been deposited in the European Nucleotide Archive (ENA) at EMBL-EBI under accession number PRJEB60226 (https://www.ebi.ac.uk/ena/browser/view/PRJEB60226).

## References

[1] *World Health Organization (WHO) Diagnostic testing for SARS-CoV-2*, https://www.who.int/publications/i/item/diagnostic-testing-for-sars-cov-2 (2020).

[2] *FIND Diagnosis for all*, https://www.finddx.org/covid-19/test-directory/ (2022).

[3] van Kasteren PB (2020). Comparison of seven commercial RT-PCR diagnostic kits for COVID-19. J. Clin. Virol..

[4] Uhteg K (2020). Comparing the analytical performance of three SARS-CoV-2 molecular diagnostic assays. J. Clin. Virol..

[5] Lowe CF (2020). Detection of low levels of SARS-CoV-2 RNA from nasopharyngeal swabs using three commercial molecular assays. J. Clin. Virol..

[6] Igloi Z (2020). Comparison of commercial realtime reverse transcription PCR assays for the detection of SARS-CoV-2. J. Clin. Virol..

[7] Kubina R, Dziedzic A (2020). Molecular and serological tests for COVID-19 a comparative review of SARS-CoV-2 coronavirus laboratory and point-of-care diagnostics. Diagnostics (Basel).

[8] Gard L, Fliss MA, Bosma F, Ter Veen D, Niesters HGM (2022). Validation and verification of the GeneFinder COVID-19 Plus RealAmp kit on the ELITe InGenius(R) instrument. J. Virol. Methods.

[9] Yang M (2022). Performance verification of five commercial RT-qPCR diagnostic kits for SARS-CoV-2. Clin. Chim. Acta.

[10] Vogels CBF (2020). Analytical sensitivity and efficiency comparisons of SARS-CoV-2 RT-qPCR primer-probe sets. Nat. Microbiol..

[11] Bogiel T, Rzepka M, Depka D (2021). Reliable diagnostics of SARS-CoV-2 infections using one- and two-gene molecular tests for a viral RNA detection-results questioning previous observations. Diagnostics (Basel).

[12] Wernike K, Keller M, Conraths FJ, Mettenleiter TC, Groschup MH, Beer M (2021). Pitfalls in SARS-CoV-2 PCR diagnostics. Transbound. Emerg. Dis..

[13] Matheeussen V (2020). International external quality assessment for SARS-CoV-2 molecular detection and survey on clinical laboratory preparedness during the COVID-19 pandemic, April/May 2020. Eurosurveillance.

[14] Vandenberg O, Martiny D, Rochas O, van Belkum A, Kozlakidis Z (2021). Considerations for diagnostic COVID-19 tests. Nat. Rev. Microbiol..

[15] Rambaut A (2020). A dynamic nomenclature proposal for SARS-CoV-2 lineages to assist genomic epidemiology. Nat. Microbiol..

[16] Callaway E (2022). Will there be a COVID winter wave? What stientists say. Nature.

[17] Xia S, Wang L, Zhu Y, Lu L, Jiang S (2022). Origin, virological features, immune evasion and intervention of SARS-CoV-2 Omicron sublineages. Signal Transduct. Target Ther..

[18] Lupala CS, Ye Y, Chen H, Su XD, Liu H (2022). Mutations on RBD of SARS-CoV-2 Omicron variant result in stronger binding to human ACE2 receptor. Biochem. Biophys. Res. Commun..

[19] Vitiello A, Ferrara F, Auti AM, Di Domenico M, Boccellino M (2022). Advances in the Omicron variant development. J. Intern. Med..

[20] Venkatakrishnan AJ (2022). On the origins of omicron’s unique spike gene insertion. Vaccines (Basel).

[21] Xu Z, Liu K, Gao GF (2022). Omicron variant of SARS-CoV-2 imposes a new challenge for the global public health. Biosaf. Health.

[22] Artik Y, Cesur NP (2022). General evaluation of COVID-19 diagnosis methods. Cohesive J. Microbiol. Infect. Dis..

[23] Artik Y, Cesur NP, Laçin NT (2022). SARS-CoV-2 mutations, diagnosis and their concern. Arch. Mol. Biol. Genet..

[24] Komurcu, S. Z. M., Artik, Y., Cesur, N. P., Kazezoglu, C., Sutasir, Y. T. Evaluation of SARS-CoV-2 patients with annual RT-PCR analysis results. *J. Clin. Exp. Investig.* **13** (2022).

[25] Artik Y, Cesur N, Kenar L, Ortatatli M (2021). Biological disasters: In the first quarter of 2021 Covid-19 overview. Afet Risk Dergisi.

[26] Komurcu SZM (2022). The evaluation of potential global impact of the N501Y mutation in SARS-COV-2 positive patients. J. Med. Virol..

[27] Artik Y (2022). In-vitro for Q-RT-PCR clinical evaluation of oscardia ledovir spray effectiveness on SARS-CoV-2 and its effective variants. Explor. Res. Hypothesis Med..

[28] Artik Y (2022). Clinic evaluation of the destrovir spray effectiveness in SARS-CoV-2 disease. Electron. J. General Med..

[29] Sule WF, Oluwayelu DO (2020). Real-time RT-PCR for COVID-19 diagnosis: Challenges and prospects. Pan. Afr. Med. J..

[30] Stahlberg A, Kubista M (2014). The workflow of single-cell expression profiling using quantitative real-time PCR. Expert Rev. Mol. Diagn..

[31] Bullard J (2020). Predicting infectious severe acute respiratory syndrome coronavirus 2 from diagnostic samples. Clin. Infect. Dis..

[32] Wolfel R (2020). Virological assessment of hospitalized patients with COVID-2019. Nature.

[33] van Kampen JJA (2021). Duration and key determinants of infectious virus shedding in hospitalized patients with coronavirus disease-2019 (COVID-19). Nat. Commun..

[34] Marot S, Calvez V, Louet M, Marcelin AG, Burrel S (2021). Interpretation of SARS-CoV-2 replication according to RT-PCR crossing threshold value. Clin. Microbiol. Infect..

[35] Platten M (2021). SARS-CoV-2, CT-values, and infectivity-conclusions to be drawn from side observations. Viruses.

[36] Singanayagam A (2020). Duration of infectiousness and correlation with RT-PCR cycle threshold values in cases of COVID-19, England, January to May 2020. EuroSurveillance.

[37] Lee RA, Herigon JC, Benedetti A, Pollock NR, Denkinger CM (2021). Performance of saliva, oropharyngeal swabs, and nasal swabs for SARS-CoV-2 molecular detection: A systematic review and meta-analysis. J. Clin. Microbiol..

[38] Perchetti GA (2020). Validation of SARS-CoV-2 detection across multiple specimen types. J. Clin. Virol..

[39] Artik Y (2022). The effect of q-RT-PCR analysis method on saline gargle samples in SARS-CoV-2 clinical diagnostic methods. Electron. J. General Med..

[40] Yilmaz Gulec E, Cesur NP, Yesilyurt Fazlioglu G, Kazezoglu C (2021). Effect of different storage conditions on COVID-19 RT-PCR results. J. Med. Virol..

